# Perspectives of Patients With Chronic Diseases on Future Acceptance of AI–Based Home Care Systems: Cross-Sectional Web-Based Survey Study

**DOI:** 10.2196/49788

**Published:** 2023-11-06

**Authors:** Bijun Wang, Onur Asan, Mo Mansouri

**Affiliations:** 1 Department of Business Analytics and Data Science Florida Polytechnic University Lakeland, FL United States; 2 School of Systems and Enterprises Stevens Institue of Technology Hoboken, NJ United States

**Keywords:** consumer informatics, artificial intelligence, AI, technology acceptance model, adoption, chronic, motivation, cross-sectional, home care, perception, perceptions, attitude, attitudes, intent, intention

## Abstract

**Background:**

Artificial intelligence (AI)–based home care systems and devices are being gradually integrated into health care delivery to benefit patients with chronic diseases. However, existing research mainly focuses on the technical and clinical aspects of AI application, with an insufficient investigation of patients’ motivation and intention to adopt such systems.

**Objective:**

This study aimed to examine the factors that affect the motivation of patients with chronic diseases to adopt AI-based home care systems and provide empirical evidence for the proposed research hypotheses.

**Methods:**

We conducted a cross-sectional web-based survey with 222 patients with chronic diseases based on a hypothetical scenario.

**Results:**

The results indicated that patients have an overall positive perception of AI-based home care systems. Their attitudes toward the technology, perceived usefulness, and comfortability were found to be significant factors encouraging adoption, with a clear understanding of accountability being a particularly influential factor in shaping patients’ attitudes toward their motivation to use these systems. However, privacy concerns persist as an indirect factor, affecting the perceived usefulness and comfortability, hence influencing patients’ attitudes.

**Conclusions:**

This study is one of the first to examine the motivation of patients with chronic diseases to adopt AI-based home care systems, offering practical insights for policy makers, care or technology providers, and patients. This understanding can facilitate effective policy formulation, product design, and informed patient decision-making, potentially improving the overall health status of patients with chronic diseases.

## Introduction

Artificial intelligence (AI) in health care represents the use of technology and machine learning algorithms to perform a range of tasks to emulate human cognition in analyzing, interpreting, and comprehending complicated medical and health care data to improve patient outcomes [1,2]. These technologies can help in decision-making and bridge some of individuals’ computational and cognitive limitations without explicit human instructions in medical practice [3-5]. AI health care applications extend beyond traditional clinical settings, integrating into direct-to-consumer (DTC) technologies. The shift in care methods from acute hospitalization to daily proactive, preventive home treatment is becoming increasingly evident [6]. Moreover, DTC technologies with AI-powered functions allow patients to participate in their own health care activities without the constraints of location and time [7]. These include health applications, wearable devices, and health monitors, which offer functionalities such as early health issue warning and prediction, social support provision, web-based communication facilitation, and delivery of personalized health advice to enhance the efficiency and effectiveness of diagnoses and treatments [8]. By integrating traditional health delivery with AI-driven services, these systems alleviate patients’ mobility and reduce the burden on the health care system [9,10]. In addition, AI-based home care systems can enhance communications and interactions between patients and health care providers. This constant connectivity allows patients to express concerns, ask questions, and receive timely feedback. Furthermore, DTC technologies promise a future where medical databases and systems can be improved based on user information and where patients are more aware of their health conditions and disease knowledge. With complex care needs and ongoing management requirements, patients with chronic diseases represent a population that stands to benefit significantly from AI-based home care systems.

Although some studies have investigated patient perceptions and attitudes toward clinical AI, very few have focused on home-based AI, especially in the context of care for patients with chronic diseases [1,5,11,12]. Additionally, nonurgent chronic conditions account for a significant portion of care needs, making it a logical population to focus on for improving AI adoption in home care settings. Therefore, exploring the factors influencing the intention of patients with chronic diseases and their interest in adopting AI-based home care systems is essential, thereby informing the design of innovative health care models for chronic conditions.

The primary objective of this paper is to identify the determinants influencing consumers’ perception of AI-based home care systems. To this end, we conducted a cross-sectional web-based survey using a hypothetical scenario and provided empirical evidence for the proposed research hypotheses. This study contributes several ways to the existing literature on AI in health care and AI-based home care systems. First, it is one of the first empirical investigations into the factors influencing the perceptions and intentions of patients with chronic diseases to adopt AI-based home care systems, diverging from the prevalent focus on the clinical performance of AI. Second, it uniquely elucidates the interplay of factors like privacy, regulation, accountability, and security in shaping the perceptions of patients with chronic diseases about usefulness and comfortability, attitudes, and adoption motivations for AI-based home care systems, and thus enriches our understanding of the complexity from social and human aspects. Third, this study adds to the theoretical understanding of technology adoption and acceptance in health care and highlights the importance of human factors in developing a framework. By shedding light on these issues, we encourage a more holistic view of users’ needs and standardize the application of AI to eliminate consumers’ concerns and increase perceived benefits. We believe this study can inform the design and implementation of AI-based home care systems that better meet the requirements and expectations of patients with chronic diseases.

## Methods

### Overview

It is critical to understand patients’ perceptions, as they directly assess the risks, benefits, and barriers involved in using these AI tools. In response, we propose a hypothetical research framework, grounded in existing literature, to explore the factors that may affect the motivations and intentions of adopting AI-based home care systems. This framework incorporates 5 constructs: privacy, accountability and security, attitude, perceived usefulness and comfortability, and motivation to adopt to fill the research gap and inform stakeholders of consumers’ needs and concerns.

### Privacy

AI-based home care systems collect and process real-time personal health data, facilitating human-computer interactions and patient health monitoring [13]. However, privacy concerns arise since users are understandably sensitive to personal data [11,14]. Privacy considerations revolve around how information is collected, stored, accessed, and shared [1,4]. These concerns could discourage individuals from sharing information and using health services, thereby hindering the widespread adoption of AI in health care delivery [15]. Beyond technology, addressing patients’ rights to oversee their data in our increasingly digital world is imperative. Crucially, regulatory compliance is situated under the umbrella of privacy because it is a crucial mechanism that enforces adherence to established data protection standards. Regulatory mandates, often developed in response to public concerns about data privacy, work to ensure that personal data are well handled [14]. Regulatory compliance is not just about legal obedience; it gives individuals a sense of assurance that their data are being managed with integrity and transparency. This underscores the pressing need for stringent regulations governing patient data acquisition, processing, and storage [16,17]. The degree of regulatory compliance and level of privacy anxiety may impact the perceived comfortability and attitude toward AI adoption. As such, our study considers 3 dimensions of privacy issues: perceived comfortability with information storage, data collection practices, and perceived regulatory compliance.

### Accountability and Security

Despite the increasing prevalence of research on AI governance issues, there is a lack of studies considering patients’ perceptions of accountability issues in this context. The lack of clear accountability for the actions of AI may create a sense of insecurity and unease for patients [18-20]. While the ongoing dialog on AI governance is becoming increasingly pertinent, there remains a notable gap in comprehending patients’ perspectives, particularly regarding the accountability and security of AI applications. The confluence of accountability and security is intentional. Accountability revolves around the notion of answerability—determining who or what entity bears the onus when AI decisions go awry. Security, on the other hand, focuses on safeguarding patient information from unwarranted access or breaches. These 2 facets are intertwined; without a transparent system of accountability, the integrity of data security is compromised. For instance, if an AI system makes a decision leading to a patient’s harm and there is no clear entity to hold accountable, it implies potential lapses in data security and the AI’s operational parameters. Navigating these complexities poses significant challenges. A lack of consensus solutions exacerbates patients’ fears about data misuse and the trustworthiness of AI systems [20]. Moreover, the inherent complexity of AI, which often results in opaque validation processes, may magnify these concerns [1,21]. Additionally, unlike humans, AI lacks subjective consciousness in its decision-making. This absence positions AI as a tool rather than an active participant with intent. Consequently, questions arise about the responsibility and accountability for AI-driven decisions, creating patient concerns about the security and reliability of relying on AI [19]. Therefore, our study explores patients’ perspectives on these concerns and examines 4 dimensions of accountability and security: data security and use, patients’ rights regarding their medical records, AI developer accountability, and physician or hospital accountability.

### Perceived Usefulness and Comfortability

Perceived usefulness, a core construct of the technology acceptance model, is crucial in evaluating technology acceptance [22,23]. In addition to perceived usefulness, this study introduces comfortability as a significant factor. We define comfortability as the degree to which patients perceive the AI-based home care systems to be comfortable for managing chronic conditions and promoting personal health status [24]. We hypothesize that patients are more likely to adopt a technology when they perceive it as beneficial (usefulness) and feel at ease and secure while using it (comfortability). In this study, these can be expressed as the degree to which the patients perceive the AI-based home care systems are useful and comfortable for managing chronic conditions and promoting personal health status [24]. Consumers evaluate usefulness based on perceived benefits and convenience [12,25] and expect enhanced communication with physicians when AI provides more information about their health status [1]. Additionally, patients expect cost reduction in long-term care while maintaining recovery quality with AI-based home care systems [26]. Furthermore, AI systems offer unlimited access to technical education and health knowledge, providing positive guidance and enhancing overall patient comfort and usefulness [23]. This measure contributes to the proposed model by capturing patients’ perception of the system from these 4 aspects: reducing health care costs, facilitating understanding of health conditions, improving communication with care providers, and educating patients about their health.

### Attitude

Successful adoption of AI-based home care systems requires an examination of patients’ attitudes and perceptions of AI [27,28]. Attitudes, which are deeply entwined with patients’ perceptions of the technology, directly influence their intention to use and motivation to accept these systems [23]. Trust is critical to patients’ attitudes toward AI, particularly when considering the balance between safeguarding personal information and receiving personalized services and treatment [29]. Moreover, patients’ comfort level with AI’s role in their treatment and their daily use frequency are also crucial in determining their attitude toward AI. If patients feel comfortable receiving the medical results from AI participated diagnoses, especially for serious diseases, a positive attitude may be fostered to alleviate doubts and distrust of the adoption. This study incorporates the attitude construct in the proposed model by examining it from 4 perspectives: attitude of daily use, attitude of AI’s future role, attitude of trust, and attitude of receiving serious diagnoses from AI.

### Hypothesis Development

In summary, we incorporate constructs drawn from the existing literature and studies, comprising 5 main constructs: privacy, accountability and security, attitude, perceived usefulness and comfortability, and motivation to adopt.

The following hypotheses are proposed to explore the key relationships between these constructs:

- H1: Privacy concern significantly impacts the perceived usefulness and comfortability from the perspective of patients with chronic diseases in adopting AI-based home care systems.
- H2: Accountability and security significantly impacts the perceived usefulness and comfortability from the perspective of patients with chronic diseases in adopting AI-based home care systems.
- H3: Privacy concern significantly impacts the attitude toward AI-based home care systems for patients with chronic diseases.
- H4: Perceived usefulness and comfortability significantly impacts the attitude toward AI-based home care systems for patients with chronic diseases.
- H5: Accountability and security significantly impacts the attitude toward AI-based home care systems for patients with chronic diseases.
- H6: Perceived usefulness and comfortability significantly impacts the motivation to adopt of AI-based home care systems for patients with chronic diseases.
- H7: The attitude of patients with chronic diseases significantly impacts the motivation to adopt of AI-based home care systems for patients with chronic diseases.

### Methodology

#### Theoretical Framework Development

**Figure 1:**
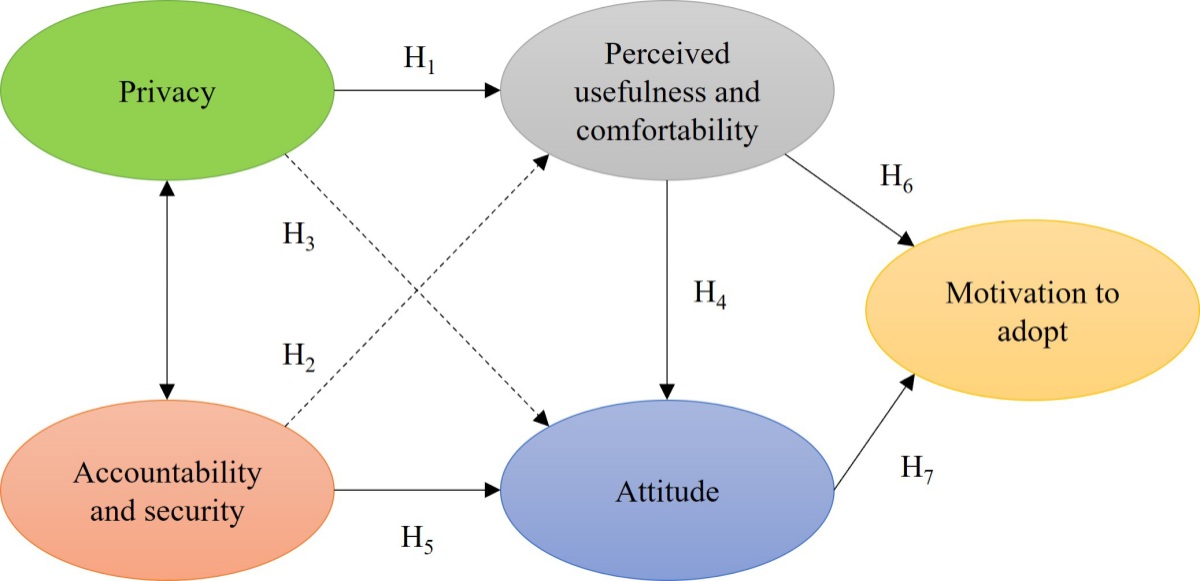
Proposed research model. H: hypothesis.

The proposed framework with corresponding research hypotheses is formulated to examine the intention of adopting AI-based home care systems from the perspective of patients with chronic diseases, as shown in Figure 1. The framework postulates that consumers’ attitudes toward adoption can be influenced by perceived usefulness and comfortability, accountability and security issues, and perceived privacy concerns. Then, the perceived usefulness and comfortability are also used as the dependent variable to explain the causal relationship with the concern about privacy and issues in accountability and security. Finally, the effect of attitude and perceived usefulness is also examined to measure the motivation to adopt. These hypotheses are fundamental in deciphering the relationships between these constructs in the AI-based home care system adoption domain.

#### Measurement

A survey-based methodology was applied to test the research hypothesis, focusing on a hypothetical AI-based home care system that patients can use for health maintenance outside hospitals. We incorporated 5 latent constructs with 17 observational variables to assess the factors influencing the perspective of patients with chronic diseases regarding the adoption of AI-based home care systems in the future. All 5 key constructs were measured using multiple items. To ensure questionnaire validation, all instruments were adopted from published research encompassing both quantitative and qualitative studies. Multimedia Appendix 1 [1,7,12,15,19,20,22,26,29-36] illustrates each construct’s derivation, items of constructs, and the source papers that influenced its formulation.

#### Data Collection

The questionnaire was distributed on Amazon Mechanical Turk (MTurk), a crowdsourcing platform known for its efficiency in individual-level data collection for health and medical domain–related social behavior studies [15,37]. MTurk can facilitate anonymous questionnaire completion without geographic or temporal constraints. All questions were formulated on 5-point Likert scale, where 1 indicates “strongly disagree” and 5 indicates “strongly agree” in the English version.

The questionnaire was divided into 3 sections. The first section consisted of an eligibility question to confirm that the respondent had one or more chronic diseases, thereby qualifying to participate in the study. Respondents were asked to consider a hypothetical AI-based home care system and answer questions using an AI-based smart device or application in their daily nonemergent care. The second section collected demographic information, including age, gender, income, education, and race. The third section consisted of 17 Likert scale questions to measure respondents’ perceptions of AI systems for managing chronic conditions at home. For instance, 1 question related to privacy asked, “I would be comfortable with the AI system keeping my medical notes, information, and history.” Meanwhile, a question aimed at understanding perceived usefulness queried, “I believe an AI-based home care device will improve the communication when I talk to my physician.” We also included a multiple-choice trap question to filter valid data for further analysis. We also provided Multimedia Appendix 2, the entire survey used to collect patient data.

Questionnaires were randomly distributed on the MTurk platform, which yielded 339 responses. We initially excluded 57 due to incorrect answers to the trap question. Subsequently, 60 duplicate responses were identified and removed to ensure data accuracy and prevent multiple submissions from the same participant. Finally, a total of 222 answers were selected for further analysis.

#### Ethical Considerations

This study was reviewed and approved by the Stevens Institute of Technology Institutional Review Board (2022-049 (N)). Participants received US $2 as compensation for survey completion.

#### Data Analysis Approach

First, we conducted a more detailed descriptive statistics for each construct and their associated variables. Then, the normality was evaluated, considering the acceptance of skewness and kurtosis value, before conducting statistical analysis. Finally, we used the structural equation model (SEM) to analyze the structural relationship for the developed framework and test the proposed hypotheses of the constructs. SEM is an exploratory multivariate data analysis technique proposed by Wold [38] and has been widely applied to multiple fields, such as business, economics, health care informatics, and information systems [23,31,32,39,40]. SEM is able to test and validate the proposed theoretical framework, offering insights into the factors influencing the motivation of patients with chronic diseases to adopt AI-based home care systems. SEM is based on a maximum likelihood algorithm that considers error terms when establishing loading factors, correlations, and other relevant observations, thus ensuring the robustness of the study results [23]. SPSS (version 27; IBM Corp) and AMOS (version 28; IBM Corp) were used for data analysis and hypothesis testing.

The goodness of fit statistics was then evaluated for the entire structural model, and the overall fit was assessed. Afterward, the internal reliability, convergent validity, and discriminant validity were tested to confirm the reliability and validity of the established SEM model. The reliability analysis was performed first to generate composite reliability and Cronbach α for internal consistency, and then confirmatory factor analysis was performed to test the convergent and discriminant validity. Finally, the research framework was tested, and the path coefficients and mediating effect were calculated.

## Results

### Participants’ Demographics

Table 1 outlines respondents’ demographic characteristics in detail. The data show a relatively balanced gender distribution, with 52.3% (n=116) males and 47.7% (n=106) females, respectively. Over half of the respondents fall within the 31-45 years age group, suggesting a concerning trend of chronic illnesses among younger individuals. The respondents’ racial composition aligns with the US Census Bureau’s report from July 2021; for instance, the percentages of self-identified White Americans from the respondents and the Census Bureau are around 72.5% and 75.8%, respectively [41]. Around 80% (n=176) of the respondents in our survey have achieved at least a bachelor’s degree, which might be indicative of a selection bias, given that MTurk platform users tend to be more educated than the average working adult population [30]. In terms of income, the majority of the respondents fall into the ranges of US $25,000-US $50,000 (n=76, 34.2%) and US $50,000-US $100,000 (n=89, 40.1%), aligning with the US median household income [42].

**Table 1:** Demographic characteristics of the respondents (N=222).

Measure			Values, n (%)
**Gender**			
	Female	116 (52.3)	
	Male	106 (47.7)	
**Race**			
	African American	10 (4.5)	
	Asian	42 (18.9)	
	Hispanic	9 (4.1)	
	White American	161 (72.5)	
**Age (years)**			
	18-30	46 (20.7)	
	31-45	118 (53.2)	
	46-60	44 (19.8)	
	>61	14 (6.3)	
**Level of education**			
	Associate degree	22 (9.9)	
	High school	24 (10.8)	
	Bachelor’s degree	109 (49.1)	
	Master’s degree	56 (25.2)	
	Doctoral degree	11 (5)	
**Household income (US $)**			
	Less than $25,000	30 (13.5)	
	$25,000-$50,000	76 (34.2)	
	$50,000-$100,000	89 (40.1)	
	$100,000-$200,000	20 (9)	
	More than $200,000	7 (3.2)	

#### Preliminary Statistical Analysis

**Figure 2:**
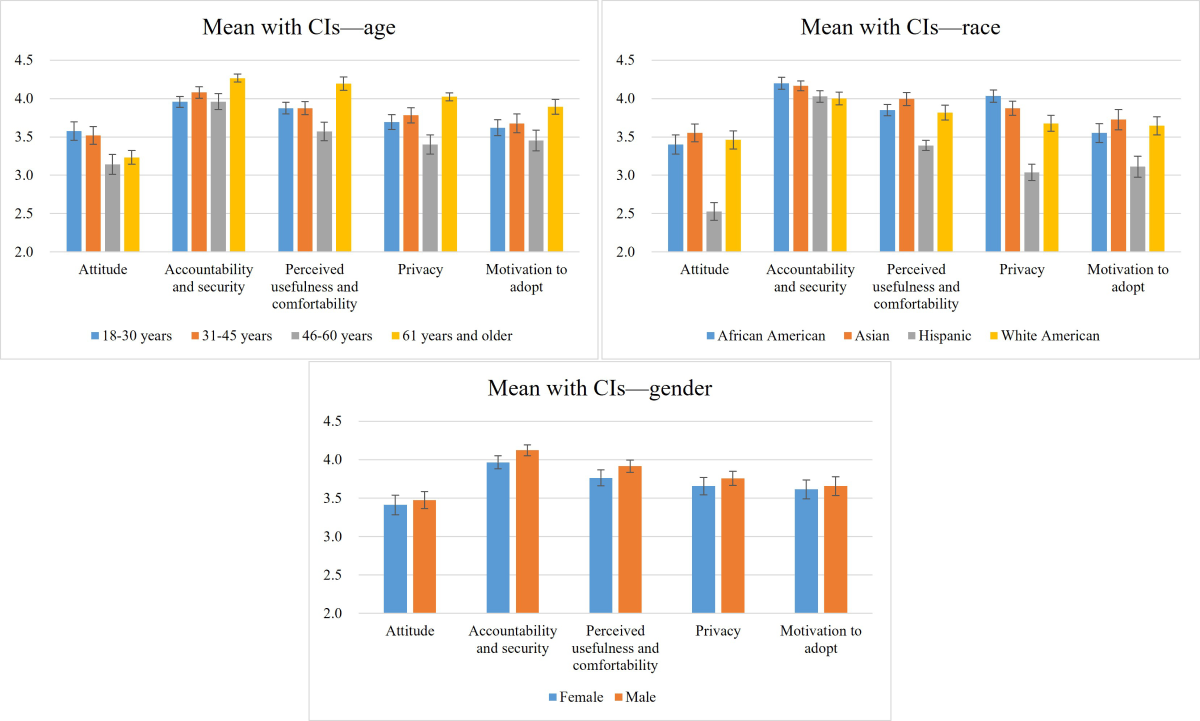
Mean and CI values associated with gender, age, and race.

Figure 2 shows all the descriptive statistics (mean and SDs) for each construct across various demographic variables, including gender, age, and race. Some of the trends are evident from the descriptions. For instance, while no significant difference exists in AI adoption perception between males and females, males slightly outscore females across all constructs. Respondents aged 60 years and older, likely due to their heightened susceptibility to chronic diseases, exhibit greater sensitivity to all types of information, reflecting their increased concern and focus on health-related information [17]. Across different race groups, Hispanic respondents express less interest in adopting AI-based home care systems, requiring more attention and communication strategies toward this minority group.

Table 2 presents descriptive statistics of the construct variables, including each construct’s mean, SD, minimum and maximum scores, skewness, and kurtosis. The perceived usefulness and comfortability received the lowest mean score (mean 3.440, SD 1.138), while attitude received the highest mean score (mean 4.042, SD 1.086). In the context of SEM, maintaining data normality is imperative to ensure an unbiased and consistent model [43]. A widely accepted guideline in SEM analysis posits that skewness and kurtosis values should ideally lie within the range of –3 to +3 [44]. All constructs’ skewness and kurtosis values are well within the accepted range. Specifically, our constructs’ skewness and kurtosis values predominantly fall within the –1 to 1 range, suggesting a well-balanced and minimally skewed data distribution. For instance, the “perceived usefulness and comfortability” construct presents a skewness of 1.138, which suggests a slight lean to the right or a minor concentration of data points on the left side of the distribution. Its kurtosis of –0.304 indicates that the data have a fairly flat peak, meaning the distribution has lighter tails and less peakness than a standard normal curve. The good skewness or kurtosis scores demonstrate the high quality and reliability of our data, which, in turn, confirms the validity of our SEM model.

**Table 2:** Descriptive statistics of constructs.

	Minimum	Maximum	Values, mean (SD)	SE	Skewness	Kurtosis
Perceived usefulness and comfortability	1	5	3.440 (1.138)	0.038	–0.705	–0.304
Privacy	1	5	3.840 (0.953)	0.032	–0.911	0.865
Accountability and security	1	5	3.701 (0.974)	0.038	–0.790	0.380
Attitude	1	5	4.042 (0.884)	0.030	–0.870	0.629
Motivation to adopt	1	5	3.644 (1.086)	0.052	–0.647	–0.271

#### Model Assessment and Evaluation

We initially checked for the statistical fit of the model. All the fit indices meet the acceptance level shown in Table 3 [45].

SEM requires an examination of convergence, content and discriminant validity, and reliability of constructs such as confirmatory factor analysis and reliability analysis [26,31]. The validity test includes both convergent and discriminant validity, while internal consistency reliability considers composite reliability and Cronbach α. Convergent validity refers to the degree to which the observation variable could effectively relate to the corresponding construct variable, while internal consistency reliability measures whether the observation variable reflects the same underlying construct variable. As shown in Table 4, all factors were in the acceptable range. Cronbach α and composite reliability values were within the acceptable 0.6-0.9 range [31,46,47]. Most factor loadings in this study were high (>0.7), with few at a medium level (>0.5), indicating adequate variance extraction from the corresponding variable [48].

Discriminant validity demonstrates that constructs should not be highly related to each other by theory, where this analysis was conducted by comparing the square root of construct’s average variance extracted and its correlation coefficients with other constructs. As shown in Table 5, the square root of each construct’s average variance extracted was greater than the correlation coefficients, indicating this study’s acceptance of discriminant validity.

**Table 3:** Research model fit.

Fit	Chi-square (*df*)	RMESA^a^	GFI^b^	AGFI^c^	CFI^d^	NFI^e^	IFI^f^
Recommended value	<3 (96)	<0.05	>0.90	>0.80	>0.90	>0.90	>0.90
Value in this study	1.503 (96)	0.049	0.935	0.887	0.972	0.926	0.973

^a^RMESA: root mean square error of approximation.

^b^GFI: goodness-of-fit index.

^c^AGFI: adjusted goodness of fit index.

^d^CFI: comparative fit index.

^e^NFI: normed fit index.

^f^IFI: incremental fit index.

**Table 4:** Result of consistency reliability.

Constructs and items			Convergent validity					Internal consistency reliability		
			Factor loading	Items reliability		AVE^a^		Cronbach α		Composite reliability
Recommended value			>0.7	>0.5		>0.5		.6-.9		0.6-0.9
**Attitude (AT)**						0.630		.828		0.869
	AT1	0.937		0.878						
	AT2	0.663		0.440						
	AT3	0.894		0.799						
	AT4	0.636		0.404						
**Perceived usefulness and comfortability (PU)**						0.503		.732		0.801
	PU1	0.771		0.594						
	PU2	0.669		0.448						
	PU3	0.742		0.551						
	PU4	0.648		0.420						
**Privacy (PR)**						0.576		.742		0.803
	PR1	0.741		0.549						
	PR2	0.807		0.651						
	PR3	0.726		0.527						
**Accountability and security (AS)**						0.600		.638		0.854
	AS1	0.897		0.805						
	AS2	0.578		0.334						
	AS3	0.721		0.520						
	AS4	0.862		0.743						
**Motivation to adopt (MA)**						0.501		.602		0.656
	MA1	0.528		0.279						
	MA2	0.851		0.724						

^a^AVE: average variance extracted.

**Table 5:** Results of discriminant validity.

	Privacy	Accountability and security	Perceived usefulness and comfortability	Attitude	Motivation to adopt
Privacy	0.759	N/A^a^	N/A	N/A	N/A
Accountability and security	0.251	0.775	N/A	N/A	N/A
Perceived usefulness and comfortability	0.635	0.304	0.709	N/A	N/A
Attitude	0.343	0.096	0.652	0.794	N/A
Motivation to adopt	0.486	0.267	0.589	0.694	0.708

^a^Not applicable.

#### Hypotheses Test Results

**Figure 3:**
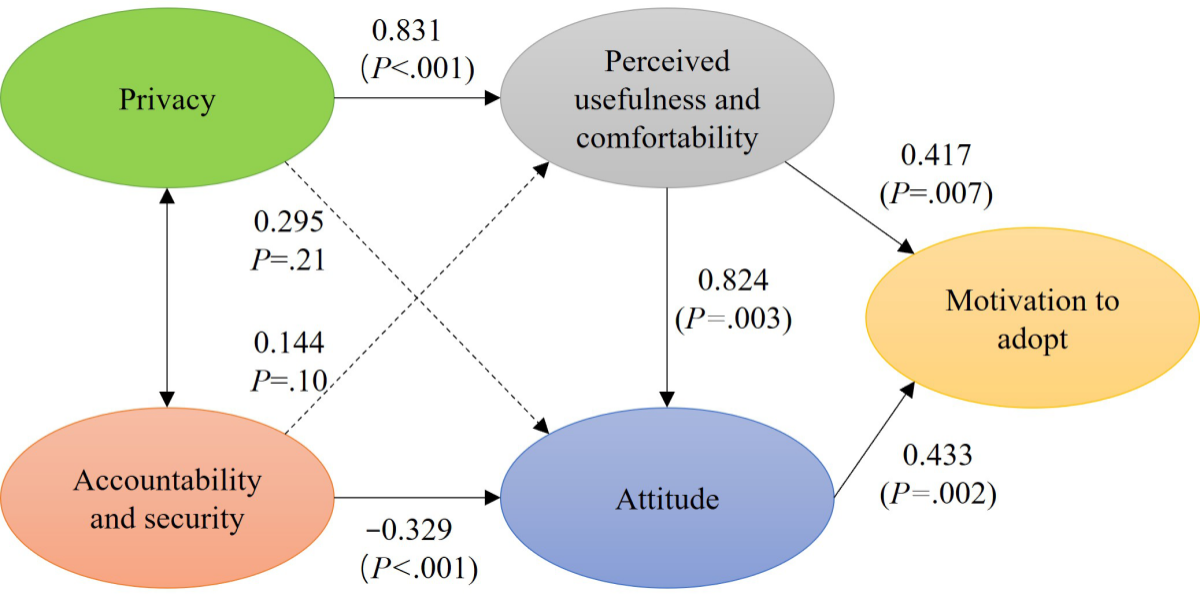
Evaluation of proposed research model.

Following satisfactory validity and reliability of the measurement model, we proceeded to hypothesis testing. Table 6 summarizes path coefficients for the hypotheses test results. The findings significantly supported 5 proposed causal relationships while 2 hypotheses were not statistically significant, as shown in Figure 3. Privacy (β=.831; *P*<.001) had significant effects on perceived usefulness and comfortability but not on attitude (β=.295; *P*=.21). Accountability and security significantly impacts attitude (β=–.329; *P*<.001) with no significant effects on perceived usefulness and comfortability (β=.144; *P*=.10). Perceived usefulness and comfortability was significantly associated with both attitude (β=.824; *P*=.003) and motivation to adopt (β=.417; *P*=.007). Attitude toward motivation to adopt was found significant (β=.433; *P*=.002). In summary, H1, H4, H5, H6, and H7 were supported, while H2 and H3 were rejected.

**Table 6:** Path coefficient result.

Hypotheses	Path	Standardized coefficient	SE	Critical ratio	*P* value	Significance
H1	PR^a^→PU^b^	0.831	0.103	8.101	<.001	Yes
H2	AS^c^→PU	0.144	0.088	1.643	.10	No
H3	PR→AT^d^	0.295	0.238	1.243	.21	No
H4	PU→AT	0.824	0.272	3.023	.003	Yes
H5	AS→AT	–0.329	0.095	–3.448	<.001	Yes
H6	PU→MA^e^	0.417	0.154	2.709	.007	Yes
H7	AT→MA	0.433	0.139	3.121	.002	Yes

^a^PR: privacy.

^b^PU: perceived usefulness and comfortability.

^c^AS: accountability and security.

^d^AT: attitude.

^e^MA: motivation to adopt.

#### Mediating Effect

In addition, 5000 resample bootstrapping procedure was applied to further analyze the structural relationships and evaluate the mediating effects. The results, including direct, indirect, and total effects, are presented in Table 7.

**Table 7:** The mediating effect for AT^a^ and MA^b^.

Path and effect		Estimate
**1: PR^c^ → PU^d^ → AT**		
	Indirect	0.637
	Direct	0.275
	Total effect	0.912
**2: AS^e^ → PU → AT**		
	Indirect	0.081
	Direct	–0.224
	Total effect	–0.143
**3: PU → AT → MA**		
	Indirect	0.406
	Direct	0.475
	Total effect	0.882

^a^AT: attitude.

^b^MA: motivation to adopt.

^c^PR: privacy.

^d^PU: perceived usefulness and comfortability.

^e^AS: accountability and security.

## Discussion

### Analysis of Results

The results supported 5 of 7 research hypotheses. The perceived usefulness and comfortability of AI-based home care systems had a direct, significant impact on patients’ motivation to adopt AI-based home care systems and an indirect influence through altering their attitudes toward AI. Besides, we observed that concerns about privacy and accountability issues may influence patients’ motivation to adopt through the usefulness and attitude toward adoption, which aligns with the previous findings [15,23]. Consumers’ privacy concerns highly impacted the perceived usefulness and comfortability (*P*<.001), corroborating earlier studies [15,33]. If AI systems were designed with adequate security and regulated to respect patients’ privacy, they perceived the system as more comfortable and usable.

Interestingly, privacy issues did not significantly affect consumers’ attitudes toward using AI-based home care systems (*P*=.21). One possible explanation could be that the direct relationship between privacy and attitude is overshadowed by other influential factors, such as perceived comfortability and perceived usefulness. The novelty of AI-based home care technology might be captivating users’ attention, causing them to prioritize its perceived benefits over potential risks. Furthermore, consumers are often known to trade off privacy for convenience, especially when the potential risks are not immediate or tangible. Given that interactions with AI are often more intuitive than the abstract concept of privacy, consumers may overlook privacy concerns until a data breach or misuse occurs [7,34]. At this stage, the perceived usefulness of AI-based home care systems temporarily outweighs privacy concerns. Additionally, the perception of privacy has been evolving rapidly in the digital age, with many consumers desensitized to data collection practices.

The issue of AI accountability is also a controversial issue in health care, as it is unclear who should hold responsibility for AI’s actions [35]. This study showed that accountability issues directly influence patients’ attitudes toward using AI-based home care systems (*P*<.001), adding unique insights to the current literature. Patients who were highly concerned about the responsibility issue tended to develop a more negative attitude toward using AI-based home systems. This suggests that clear regulations around responsibility would be enacted to enhance the usage confidence [15], which is supported by the early findings related to technology adoption in health care [49,50]. However, we did not find a significant effect of accountability on perceived usefulness and comfortability (*P*=.10). One possible explanation is that while accountability is crucial for trust-building, its impact is perhaps more indirect in nature. Patients may conceptualize accountability as a macro-level concern, pertinent mainly to regulators and AI developers. Thus, it may not directly translate to their perceptions about how useful or comfortable an AI system is for their day-to-day needs. This suggests that even though patients desire a clear understanding of who is accountable during system errors, they may not see these concerns as directly affecting the immediate advantages or their perception of the utility and comfort of AI-based home care systems. Moreover, it is possible that patients assume that once the technology has been approved and is available on the market, the accountability issues have been duly addressed by relevant authorities [19]. Hence, while accountability concerns can affect their general attitude, it does not seem to permeate their evaluation of the system’s practicality or convenience. For a comprehensive embrace of AI systems in home care, it is paramount that governance bodies understand these nuanced reactions to accountability, recognizing that a perceived lack of it could impair patient trust [35].

On the other hand, patients with chronic diseases desire AI to offer convenience and usefulness in health management at home rather than going to clinics with long waiting times [26]. Consistent with prior research [25,51], this study reaffirmed that the motivation of patients to adopt AI-based home care systems stems from the perceived usefulness and comfortability of these systems (*P*=.007) as well as the attitudes toward the adoption (*P*=.002). Furthermore, we also concluded that perceived usefulness and comfortability was strongly associated with the performance expectancy on attitude (*P*=.003), consistent with the previous study [23]. Thus, for potential consumers with chronic diseases, recognizing the practicality of AI-related systems fosters positive attitudes toward acceptance, enhancing adoption motivation [31,52].

### Implications for Care or Technology Providers

As the developers and distributors of AI-based home care systems, care or technology providers have much earlier access to the system than the end-user patients. It has always been a challenge to develop AI-based home care systems that meet the majority of end users’ expectations. However, they can still proactively anticipate and address user needs, which is crucial in facilitating user adoption and satisfaction. In this context, this study offers valuable implications.

While it is widely acknowledged that any novel technology should provide comfort and use, this study suggests that user’s trust in the systems’ functionality and ethical integrity can also positively impact adoption decisions [53]. The care or technology providers are responsible for developing a reliable, interpretable system to alleviate user anxiety. Since the entire AI process is similar to a black box, care or technology providers should work to validate the AI algorithms and present them more understandably if needed [10]. This implies that care or technology providers should design and implement secure data storage and transmission mechanisms, making it transparent and clear for users how their data are used and protected. Care or technology providers should also empower users with control over their own data, allowing them to view, correct, and delete their data as needed [40].

Importantly, the primary role of AI at this stage is not to replace but to supplement and enhance primary care. The design of AI systems should be patient-centric, taking into account the diverse needs of individuals with chronic conditions. A system customizable to various health conditions, lifestyles, and user preferences can foster a sense of personalization and thus promote engagement and long-term use [53]. By providing tools with clear, concise, and user-friendly instructions, AI can guide patients to improve doctor-patient communication and make care delivery more cost-effective, resulting in efficient doctor-AI-patient interactions.

Moreover, comprehensive and straightforward education and ongoing support should be personalized based on the individual user’s health condition and learning capability [54]. It is important that patients understand their role and have the necessary information to make informed choices rather than being passive AI recipients. Guidelines in this regard can increase patient interest in AI use and their adoption intentions. The regular feedback from patients is also crucial for continuous improvement. Providers can leverage AI technologies to capture real-time user feedback and use these data to refine the system continuously.

### Implications for Policy Makers

AI’s emergence in health care has not been met with timely policy adaptations, as technology often outpaces regulatory responses [1,19]. This study has investigated patients’ perceptions of the regulation and governance to provide insights to policy makers for better adaptation in AI-based home care.

One of the biggest concerns patients have is about the management of their medical data by AI-based home care systems. Concerns primarily revolve around data sharing, exchange, and their ethical implications. These emerging issues challenge traditional health care ethics, requiring policy makers to balance the potential benefits against patients’ privacy rights. To address these challenges, policy makers are advised to clearly define the legal and ethical boundaries of data collection, storage, use, and sharing. Establishing and enforcing standards and certification mechanisms for AI systems’ safety, effectiveness, and compliance would be prudent. Policy makers must ensure that patients are fully informed about the data that are being collected, why it is being collected, and how it will be used, and that they can make informed decisions when using AI-based home care systems.

Moreover, accountability in the current governance system is unclear, particularly in defining AI involvement in decision-making for care delivery and the extent of responsibility for biases and errors. Any unclear and opaque responsibility delineation could undermine patients’ trust and further impact perceived comfortability [19]. A clear accountability guideline should address issues such as who is responsible for the AI recommendation errors and how to handle bias results in unfair treatment or outcomes for certain groups of patients. In such contexts, while AI developers must uphold and strive for the highest precision standards, the primary accountability for the decision-making process would logically reside with the health care professionals. On the other hand, in situations where AI systems are designed to play a more independent role, particularly in remote patient monitoring setups without immediate human oversight, the responsibility might predominantly fall on the AI providers because their systems function autonomously without human checks. Establishing clear guidelines in these areas would likely enhance patients’ trust in and willingness to adopt AI solutions.

### Limitation and Future Study

This study has a few limitations. First, this study used a sample from a crowdsourcing marketplace in the United States. There is a challenge in verifying the authenticity of the health conditions claimed by respondents. Moreover, using MTurk may have introduced a certain degree of sample bias, limiting the generalizability of our findings. To ensure the accuracy of our data, we initially sampled over 300 individuals, though we acknowledge the inherent limitations in fully verifying the chronic condition status of respondents. Specifically, a considerable proportion of our respondents were relatively young, ranging from 31 to 45 years old, and were well-educated, with approximately 80% (n=176) possessing bachelor’s degrees or advanced degrees. This demographic distribution may not represent the typical profile of patients with chronic diseases, who are often older and display a broader range of education levels [16,17]. Such discrepancy highlights the potential anomaly in our sampling strategy and suggests caution in interpreting results with broader, more diverse populations. Furthermore, some patients may experience multiple chronic conditions simultaneously. This complexity could have significant implications on the required health care resources and the patients’ attitudes toward AI-based home care systems. Future studies could aim to understand patients’ diverse health conditions and varied health care demands to deepen our understanding of patients’ acceptance of AI-based home care systems. However, this limitation does not detract from the significance and originality of this work within the scope of the defined sample.

Moreover, in future research, we plan to incorporate more rigorous verification mechanisms, such as requiring medical documentation or collaborating with health care institutions, to ensure the authenticity of participants’ health conditions. This will provide a more robust data collection foundation and further strengthen our research outcomes’ validity. Future research could also aim to explore more diverse and representative patient samples, considering the variations in backgrounds and health care demands.

### Conclusions

AI-based home care systems are a promising development in health care, potentially improving the delivery and accessibility of care for patients with chronic diseases. Our findings indicate that patients have an overall positive perception of AI-based home care systems, and their motivation to adopt such systems is significantly influenced by the perceived usefulness and comfortability and their attitude toward use. However, persistent concerns around privacy and accountability underscore the need for improved data management and comprehensive regulations. This study provides invaluable insights for a range of stakeholders, including policy makers, health care providers, and patients, to effectively and ethically use AI-based home care systems. As the field evolves, research should continue to refine and expand upon these insights, enabling us to leverage AI’s potential to enhance health care outcomes fully.

## Appendix

Multimedia Appendix 1 Operationalization of constructs and items.

Multimedia Appendix 2 Survey questions.

## Abbreviations

AI: artificial intelligence
DTC: direct-to-consumer
MTurk: Mechanical Turk
SEM: structural equation model
