# Supplementary material for: Perspectives of Patients With Chronic Diseases on Future Acceptance of AI–Based Home Care Systems: Cross-Sectional Web-Based Survey Study
Source: JMIR Hum Factors. 2023 Nov 6;10:e49788. doi: 10.2196/49788 (PMC10660233; doi:10.2196/49788)
Supplement: Multimedia Appendix 1 [file humanfactors_v10i1e49788_app1.docx]

## Multimedia Appendix 1. Operationalization of constructs and items.

| Constructs | Items | Description | Sources |
| --- | --- | --- | --- |
| **Attitude** | AT1 | To what extent the chronic patients are willing to use AI-based home care system daily | [1,12,22,29,32,33,36] |
|  | AT2 | To what extent the chronic patients believe AI-based home care system will in place of the primary care doctor |  |
|  | AT3 | The degree of trustworthy to AI-based home care system |  |
|  | AT4 | The extent to which chronic patients are willing to receive serious medical diagnosis results from AI |  |
|  |  |  |  |
| **Perceived Usefulness and Comfortability** | PU1 | The degree of perceived usefulness and benefit that AI could reduce the costs of healthcare for chronic patients | [1,7,19,26,30,31,36] |
|  | PU2 | The degree of perceived usefulness that AI may facilitate the understanding of chronic patients’ health conditions |  |
|  | PU3 | The degree of perceived usefulness that AI may improve the communication efficiency and effectiveness with care providers |  |
|  | PU4 | The degree of perceived usefulness that chronic patients can be educated or trained to be better know their own health conditions by AI |  |
|  |  |  |  |
| **Privacy** | PR1 | The degree of comfortability of keeping medical notes, information and history by AI system | [1,15,29,32] |
|  | PR2 | The degree of comfortability of collecting data and personal information by AI system |  |
|  | PR3 | The extent to which chronic patients believe that mandated and adequate regulation will protect their privacy and promote the interests of AI adoption |  |
|  |  |  |  |
| **Accountability and Security** | AS1 | The extent to which chronic patients concern about the information security the final usage of personal data and information collected by an AI-based home care device | [1,15,19,20,34,35] |
|  | AS2 | The extent to which chronic patients concerns about the right to access, store and delete their medical records collected by the AI-based home care device |  |
|  | AS3 | The extent to which chronic patients believe physicians/hospitals should be held the accountability or liability for errors caused by AI |  |
|  | AS4 | The extent to which chronic patients believe AI companies/developers should be held the accountability or liability for errors caused by AI |  |
|  |  |  |  |
| **Motivation to adopt** | MA1 | The degree of interests to use AI-based home care system to manage chronic conditions and overall health in the future | [26,30,31,32,33] |
|  | MA2 | The extent to which chronic patients are motivated to believe AI-based home care system will improve the overall health |  |
