# Supplementary material for: Perspectives of Patients With Chronic Diseases on Future Acceptance of AI–Based Home Care Systems: Cross-Sectional Web-Based Survey Study
Source: JMIR Hum Factors. 2023 Nov 6;10:e49788. doi: 10.2196/49788 (PMC10660233; doi:10.2196/49788)
Supplement: Multimedia Appendix 2 [file humanfactors_v10i1e49788_app2.docx]

Patient's Perception and Opinions of AI- based Applications in Healthcare System

This survey is designed to understand the perception of chronic patients toward AI-based technology in the healthcare system. We are reaching out to participants with chronic conditions. You are eligible for the survey if you are in such conditions listed below.

Otherwise, please end the survey now. Thank you very much for your understanding and participation.

* Indicates required question

1. Do you have at least one of the listed conditions below? Check all that apply. *

(Please note: You are eligible for the survey if you are in such conditions. Otherwise, please end the survey now.Thank you for your understanding).

*Check all that apply.*

Diabetes Obesity

High blood pressure (Hypertension) High cholesterol

Depression

Tobacco Use and Related Conditions Arthritis

Heart diseases Stroke

Eating disorders Asthma

Oral Health

Alzheimer’s disease and dementia Osteoporosis

Chronic kidney disease Cancer

Cystic fibrosis

Chronic obstructive pulmonary disease (COPD) Reflex Sympathetic Dystrophy (RSD) Syndrome

Other:

Demographic Questions

2. What gender do you identify yourself with? *

*Mark only one oval.*

Female Male

Prefer not to answer

Other:

3. Which race do you identify yourself with? *

*Mark only one oval.*

African American White American Hispanic

Asian

Prefer not to answer

Other:

4. Which age group do you identify yourself within? *

*Mark only one oval.*

18-30 years

31-45 years

46-60 years

61 years and above Prefer not to answer

5. What is the highest degree or level of education you have completed? *

*Mark only one oval.*

High School Associate Degree Bachelor's Degree Master's Degree Doctoral Degree Prefer not to answer Other:

6. What is your annual household income? *

*Mark only one oval.*

Less than $25,000

$25,000 - $50,000

$50,000 - $100,000

$100,000 - $200,000

More than $200,000 Prefer not to say

Questions on General Perception of Artificial Intelligence

Definition of AI: Artificial intelligence (AI) is an umbrella term to describe the application of machine learning (ML) algorithms and other cognitive technologies. In the simplest sense, AI refers to computers and other machines that mimic human cognition and are capable of learning, thinking, and making decisions or taking actions. AI in healthcare, then, is the use of technology or machine in diagnosis, treatment, prevention, surgery, delivery of information, monitoring, analysis, and act on medical data with the goal of a particular outcome. These technologies can be applied in healthcare and medical settings to help the decision-making process with doctor-patient-AI interactions, where doctors and AI systems might have the same suggestion or opposite suggestions.

General Question of the AI-based Tools in Healthcare

In this part of the survey, please respond the following questions based on your overall perception of Artificial Intelligence (AI) System in Healthcare with best of your ability and knowledge.

7. How do you rate your knowledge about artificial intelligence (AI) in general? *

*Mark only one oval.*

Not Familiar Somewhat Familiar Familiar

Very Familiar Expert

8. How do you rate your knowledge about artificial intelligence (AI) in medical *

practice?

*Mark only one oval.*

Not Familiar Somewhat Familiar Familiar

Very Familiar Expert

9. I think AI systems would be used by doctors in decision-making for my health. *

*Mark only one oval.*

Strongly Disagree Disagree

Neutral Agree

Strongly Agree

10. I am comfortable if the AI is totally in automation rather than controlled by *

health professionals.

*Mark only one oval.*

Strongly Disagree Disagree

Neutral Agree

Strongly Agree

11. I think AI in healthcare will be the general trend in the future. *

*Mark only one oval.*

Strongly Disagree Disagree

Neutral Agree

Strongly Agree

12. I think AI will replace medical professionals in the future. *

*Mark only one oval.*

Strongly Disagree Disagree

Neutral Agree

Strongly Agree

13. I think AI may be misused in my treatment that may lead to some negative or *

unwanted outcomes.

*Mark only one oval.*

Strongly Disagree Disagree

Neutral Agree

Strongly Agree

14. I think AI outcomes might have bias due to the selection of algorithms, *

training set/data or targeted people.

*Mark only one oval.*

Strongly Disagree Disagree

Neutral Agree

Strongly Agree

15. Has any of the doctor/hospital ever used AI-based system for your care? *

*Mark only one oval.*

Yes No

I do not know

15 Continued. If Yes, please write it down. If No or I do not know, please leave it blank.

16. I believe that the AI system should be approved by related authorities before *

its use in hospitals.

*Mark only one oval.*

Strongly Disagree Disagree

Neutral Agree

Strongly Agree

17. Overall, I would trust AI in my treatment. *

*Mark only one oval.*

Strongly Disagree Disagree

Neutral Agree

Strongly Agree

Open-Ended Questions

The questions below are open-ended. Please write in the box.

18. Will it concern you whether AI is used in your treatment/care? *

*Mark only one oval.*

Yes No

18 Continued. Will it concern you whether AI is used in your treatment/care? Why/Why not? Please explain/write down.

19. In your opinion, what are the factors which can motivate or prevent you to *

use the AI based systems or accept/deny AI systems to manage your overall health or involve in the treatment process? Please explain/write down.

20. What are the factors which can affect your trust in AI systems? Please *

explain/write down.

21. In your opinion, what might be the role of government or any other * concerned authority in controlling and regulating AI systems? Please explain/write down.

22. If you have been given an opportunity to advise the AI-based system * developer for patient care, what would be the main features you want to see? Please explain/write down.

23. In the case of wrong decision which harms patient. Who would be held * accountable or responsible: the doctor or the AI system? How does this "accountability or responsibility" issue impact your decision to accept AI to be

used in your treatment and care? Please explain/write down.

24. In the case of different recommendations between your doctor and AI *

system, which one would you prefer? And Why? Please explain/write down.

Specific Questions

In this part, we describe a specific AI system that will be used by you at home to manage your chronic condition and overall health. You will respond questions for this specific AI-based home care device.

AI-based Home Care System

In this part of the survey, you will respond to the questions below based on a hypothetical AI system. This is called the AI-based home care system, which can remotely help chronic patients monitor their health conditions anywhere and anytime outside the hospital. Please consider you were given an AI-based smart device or app installed on your smartphone which performs real- time patient monitoring and virtual assistance. For example, the AI system will collect and track your health data daily, make suggestions, and transmit them to your physician or care professionals. The AI systems may make suggestions and predict outcomes based on data collected from many chronic patients from different backgrounds. Please respond to the questions to the best of your ability.

25. I would be comfortable with using AI-based home care device for my daily *

care.

*Mark only one oval.*

Strongly Disagree Disagree

Neutral Agree

Strongly Agree

AT1: To what extent the chronic patients are willing to use AI-based home care system daily

26. I believe the AI-based home care device will reduce my medical expense. *

*Mark only one oval.*

Strongly Disagree Disagree

Neutral Agree

Strongly Agree

PU1: The degree of perceived usefulness and benefit that AI could reduce the costs of healthcare for chronic patients

27. I believe a too complicated AI-based home care device will prevent me from *

using it .

*Mark only one oval.*

Strongly Disagree Disagree

Neutral Agree

Strongly Agree

28. I would believe AI-based home care device will replace *

my primary care doctor.

*Mark only one oval.*

Strongly Disagree Disagree

Neutral Agree

Strongly Agree

AT2: To what extent the chronic patients believe AI-based home care system will in place of the primary care doctor

29. I would be comfortable receiving serious medical diagnosis results (i.e., *

Cancer) from an AI-based home care device.

*Mark only one oval.*

Strongly Disagree Disagree

Neutral Agree

Strongly Agree

AT4: The extent to which chronic patients are willing to receive serious medical diagnosis results from AI

30. I would be comfortable with the AI based home system collecting data from *

me.

*Mark only one oval.*

Strongly Disagree Disagree

Neutral Agree

Strongly Agree

PR2: The degree of comfortability of collecting data and personal information by AI system

31. I would be comfortable with the AI system keeping my medical notes, *

information and history.

*Mark only one oval.*

Strongly Disagree Disagree

Neutral Agree

Strongly Agree

PR1: The degree of comfortability of keeping medical notes, information and history by AI system

32. I think sharing my information with the AI-based home care device will put *

my personal information at risk.

*Mark only one oval.*

Strongly Disagree Disagree

Neutral Agree

Strongly Agree

33. I think I should be aware of the final usage of my personal and health data *

collected by an AI-based home care device.

AS1: The extent to which chronic patients concern about the

*Mark only one oval.*

Strongly Disagree Disagree

Neutral Agree

Strongly Agree

information security, the final usage of personal data and information collected by an AI-based home care device

34. I believe using AI-based home care system may facilitate the understanding *

of my own health conditions.

*Mark only one oval.*

Strongly disagree Disagree

Neutral Agree

Strongly Agree

PU2: The degree of perceived usefulness that AI may facilitate the understanding of chronic patients’ health conditions

35. I think I should have the right to access, store and delete my own medical *

records collected by the AI-based home care device.

*Mark only one oval.*

AS2: The extent to which chronic patients concerns about the right to access, store and delete their medical records collected by the AI-based home care device

Strongly Disagree Disagree

Neutral Agree

Strongly Agree

36. I would be willing to get a training on how to use AI-based home care system *

in a better way and know better about my own health.

*Mark only one oval.*

Strongly Disagree Disagree

Neutral Agree

Strongly Agree

PU4: The degree of perceived usefulness that chronic patients can be educated or trained to be better know their own health conditions by AI

37. I believe physicians/hospitals should be held accountable or liable for patient *

outcomes when errors result from the use of the AI-based home care device.

*Mark only one oval.*

Strongly Disagree Disagree

Neutral Agree

Strongly Agree

AS3: The extent to which chronic patients believe physicians/hospitals should be held the accountability or liability for errors caused by AI

38. I believe AI company/developer should be held accountable or liable for *

patient outcomes when errors result from the use of the AI-based home care

device.

*Mark only one oval.*

Strongly Disagree Disagree

Neutral Agree

Strongly Agree

AS4: The extent to which chronic patients believe AI companies/developers should be held the accountability or liability for errors caused by AI

39. I am aware of who is responsible when errors result from the use of the AI- *

based home care device.

*Mark only one oval.*

Strongly Disagree Disagree

Neutral Agree

Strongly Agree

40. I think the mandated and adequate regulations will promote my interest and *

mitigate my privacy concern in using AI-based home care system.

*Mark only one oval.*

Strongly Disagree Disagree

Neutral Agree

Strongly Agree

PR3: The extent to which chronic patients believe that mandated and adequate regulation will protect their privacy and promote the interests of AI adoption

41. To show you are paying attention, please answer the question: which of the *

following is not a type of public transportation?

*Mark only one oval.*

Bus Airplane Apartment Train Subway

42. I think the attitude, perception or behavior of people around me would affect *

my acceptance and use of AI-based home care devices.

*Mark only one oval.*

Strongly Disagree Disagree

Neutral Agree

Strongly Agree

43. Overall, would you be interested in using an AI-based home care device to *

manage your chronic condition and overall health in the future?

*Mark only one oval.*

Very Interested Somewhat Interested Neutral

Somewhat not Interested Not Interested at all

MA1: The degree of interests to use AI-based home care system to manage chronic conditions and overall health in the future

44. Overall, I would trust AI based home care device. *

*Mark only one oval.*

Strongly Disagree Disagree

Neutral Agree

Strongly Agree

AT3: The degree of trustworthy to AI-based home care system

45. I believe AI-based home care system will improve my overall health. *

*Mark only one oval.*

Strongly Disagree Disagree

Neutral Agree

Strongly Agree

MA2: The extent to which chronic patients are motivated to believe AI- based home care system will improve the overall health

46. I think the AI-based home care device would improve my communication efficiency and effectiveness with my doctor.*

*Mark only one oval.*

Strongly Disagree Disagree

Neutral Agree

Strongly Agree

PU3: The degree of perceived usefulness that AI may improve the communication efficiency and effectiveness with care providers

47. What might be the factors which affect your decision to use or not to use AI- *

based home care system. Please explain/write down.

48. In your opinion, what might be the role of government or any other *

concerned authority in controlling AI-based home care device? Please explain/write down.

49. If you have been given an opportunity to advise the developer of AI-based * home care device for patient care, what would be the main features you want to see? Please explain/write down.

Thank you for taking the time to complete the survey and submitting it back to us. Please paste the survey code: HAPPY2022 to Mturk survey page to ensure you can receive the payment.

Survey code: HAPPY2022

1. What is your worker's ID? *

This content is neither created nor endorsed by Google.

[Forms](https://www.google.com/forms/about/?utm_source=product&utm_medium=forms_logo&utm_campaign=forms)
